# Left atrial voltage, circulating biomarkers of fibrosis, and atrial fibrillation ablation. A prospective cohort study

**DOI:** 10.1371/journal.pone.0189936

**Published:** 2018-01-02

**Authors:** Gordon A. Begg, Rashed Karim, Tobias Oesterlein, Lee N. Graham, Andrew J. Hogarth, Stephen P. Page, Christopher B. Pepper, Kawal Rhode, Gregory Y. H. Lip, Arun V. Holden, Sven Plein, Muzahir H. Tayebjee

**Affiliations:** 1 Department of Cardiology, Leeds General Infirmary, Leeds, United Kingdom; 2 Leeds Institute of Cardiovascular and Metabolic Medicine, University of Leeds, Leeds, United Kingdom; 3 Department of Biomedical Engineering, King’s College, London, United Kingdom; 4 Institute of Biomedical Engineering, Karlsruhe Institute of Technology, Karlsruhe, Germany; 5 University of Birmingham Institute of Cardiovascular Science, City Hospital, Birmingham, United Kingdom; 6 Aalborg Thrombosis Research Unit, Department of Clinical Medicine, Aalborg University, Aalborg, Denmark; 7 School of Biomedical Sciences, University of Leeds, Leeds, United Kingdom; University of Miami School of Medicine, UNITED STATES

## Abstract

**Aims:**

To test the ability of four circulating biomarkers of fibrosis, and of low left atrial voltage, to predict recurrence of atrial fibrillation after catheter ablation.

**Background:**

Circulating biomarkers potentially may be used to improve patient selection for atrial fibrillation ablation. Low voltage areas in the left atrium predict arrhythmia recurrence when mapped in sinus rhythm. This study tested type III procollagen N terminal peptide (PIIINP), galectin-3 (gal-3), fibroblast growth factor 23 (FGF-23), and type I collagen C terminal telopeptide (ICTP), and whether low voltage areas in the left atrium predicted atrial fibrillation recurrence, irrespective of the rhythm during mapping.

**Methods:**

92 atrial fibrillation ablation patients were studied. Biomarker levels in peripheral and intra-cardiac blood were measured with enzyme-linked immunosorbent assay. Low voltage (<0.5mV) was expressed as a proportion of the mapped left atrial surface area. Follow-up was one year. The primary endpoint was recurrence of arrhythmia. The secondary endpoint was a composite of recurrence despite two procedures, or after one procedure if no second procedure was undertaken.

**Results:**

The biomarkers were not predictive of either endpoint. After multivariate Cox regression analysis, high proportion of low voltage area in the left atrium was found to predict the primary endpoint in sinus rhythm mapping (hazard ratio 4.323, 95% confidence interval 1.337–13.982, p = 0.014) and atrial fibrillation mapping (hazard ratio 5.195, 95% confidence interval 1.032–26.141, p = 0.046). This effect was also apparent for the secondary endpoint.

**Conclusion:**

The studied biomarkers do not predict arrhythmia recurrence after catheter ablation. Left atrial voltage is an independent predictor of recurrence, whether the left atrium is mapped in atrial fibrillation or sinus rhythm.

## Introduction

Atrial fibrillation remains a significant cause of morbidity. For many patients, AF ablation has been shown to be a successful treatment, however approximately one third of patients undergoing the procedure will experience recurrence of AF, even after multiple procedures.[1] This figure may be as high as 50% for those with persistent AF.[2] Better patient selection may be one method of improving upon this.

Left atrial (LA) fibrosis is associated with AF, and with AF recurrence after ablation.[3] Therefore, pre- and intra-procedural assessment of fibrosis may inform selection for first-time, or subsequent, ablation.

Fibrosis involves numerous biochemical pathways, and component compounds of those pathways enter the bloodstream.[4] Such compounds, for example products and mediators of collagen turnover, can therefore be used as circulating biomarkers. There is conflicting evidence regarding their utility in predicting AF recurrence after ablation, however if such utility could be established they would be attractive to clinicians as a minimally invasive method of improved patient selection.[4]

After reviewing previous research in this field, we selected four biomarkers for study: Fibroblast growth factor 23 (FGF-23), galectin-3 (gal-3), type III procollagen N terminal peptide (PIIINP) and type I collagen C terminal telopeptide (ICTP). FGF-23 has not been studied in this context previously.

Low voltage areas identified during endocardial mapping of the LA can indicate increased likelihood of AF recurrence after ablation.[5] These low voltage areas are thought to be associated with left atrial ‘scar’ or fibrosis. In previous studies assessing voltage as a predictor of rhythm outcome, the LA has been mapped while in sinus rhythm (SR).[5] This is not necessarily representative of usual clinical practice, as patients in AF at the time of ablation are not routinely cardioverted before anatomical mapping.

We hypothesized that the predictive effect of voltage mapping would be present in those patients who were mapped in both AF and in SR, and that increased levels of the selected biomarkers would predict AF recurrence after ablation.

## Methods

Ethical approval was granted by the National Research Ethics Service Committee—Leeds West (ref. 13/YH/0349). Written informed consent was obtained from all patients. At a single institution, between September 2014 and August 2015, all patients undergoing first-time left atrial ablation for paroxysmal, persistent, or long-standing-persistent AF were screened. Patients with systemic inflammatory disease, recent or active malignancy, severe kidney disease (eGFR < 30 ml/min/1.73m^2^), previous AF ablation, or collagen disease were excluded.

All participants underwent trans-thoracic echocardiography, by a single operator with over 5 years’ experience. Images were obtained according to British Society of Echocardiography guidelines, and normal ranges for measurements were defined in accordance with these guidelines.[6] Atrial and ventricular volume measurements were acquired using Simpson’s biplane method. Antero-posterior LA diameter was measured from the 2D parasternal long-axis view. Atrial measurements were acquired at the end of ventricular systole.

Radiofrequency (RF) ablation was performed according to the 2012 international consensus statement.[7] Under general anesthetic, or conscious sedation with local anaesthetic, venous access was obtained via the right and left femoral veins. Intravenous heparin was administered to maintain activated clotting time >300s. After trans-septal puncture, irrespective of rhythm, LA bipolar voltages were recorded using a high density multi—electrode circular EP mapping catheter and 3D mapping system (Lasso/CARTO, Biosense-Webster, or Optima/EnSite Velocity, St. Jude Medical). The mapping catheter was systematically moved across the entire LA endocardium, with a minimum mapping time window for any given electrode position of 2 seconds. This minimum mapping time window was to account for any variation in voltage over time, particularly in those patients who were in AF during mapping. The lowest number of points acquired for the LA endocardial map was 864. Patients in AF were not cardioverted to sinus rhythm prior to mapping.

Right and left atrial mean pressures were measured by transduction of the LA sheath before and after trans-septal puncture. Blood was aspirated from the femoral vein sheath, and via long sheath, the right and left atria, and coronary sinus ostium for later analysis. RF energy was then applied in order to perform wide-area circumferential ablation to achieve pulmonary vein isolation (PVI). A contact force-sensing ablation catheter was used. In non-paroxysmal AF, linear ablation and/or ablation of complex fractionated electrograms were carried out at the operator’s discretion. Successful PVI was confirmed in all patients by demonstration of exit and entry block. Repeat ablation was performed if the patient had a symptomatic recurrence of arrhythmia after the blanking period, and the patient and clinician felt that these on-going symptoms justified further intervention. In repeat procedures, PVI was re-checked and, if veins were re-connected, targeted ablation was performed to achieve isolation. Further linear or substrate—targeting ablation was carried out at the discretion of the operator. Symptomatic recurrences of arrhythmia during the blanking period were treated with external electrical cardioversion.

**Fig 1:**
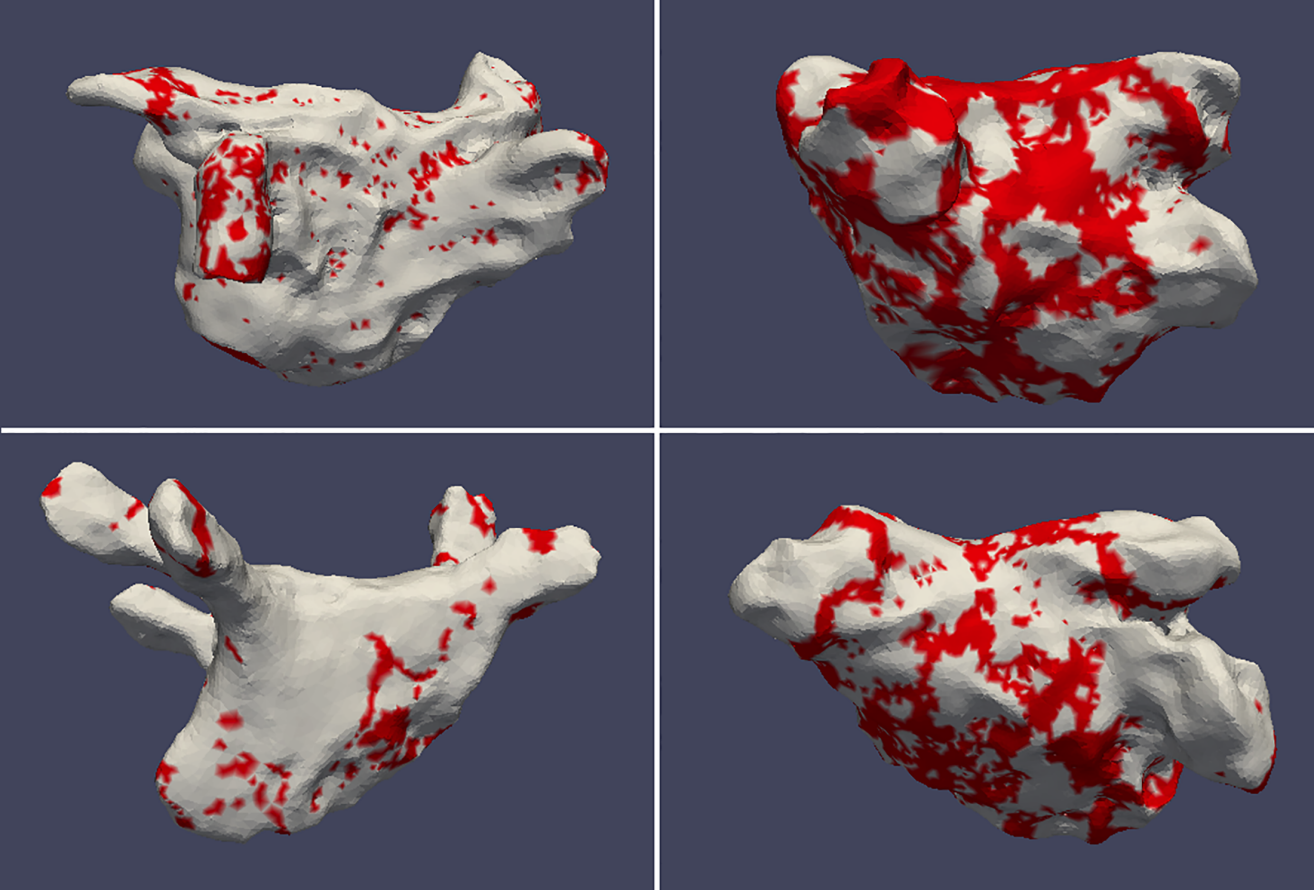
Representative LA voltage maps of 4 separate patients. Top row—patients mapped in AF. Bottom row—patients mapped in SR. Red areas represent <0.5mV. Left column—<30% low voltage, right column >30% low voltage.

Voltage maps created during the first ablation procedure were exported from the mapping system, according to the manufacturer’s instructions, as raw data. This was then reformatted to enable reconstruction, manipulation and analysis of the voltage maps in 3D image analysis software according to previously published methods.[8] The pulmonary veins, left atrial appendage, and mitral valve surface were removed from the individual left atrial anatomical reconstructions. The resulting atrial reconstruction therefore comprised a geometric representation of the LA endocardium with embedded surface voltage data. The proportion of the mapped LA surface exhibiting low voltage (<0.5mV) was expressed as a percentage of the overall mapped LA surface area, not including the anatomical structures removed as described. Fig 1 shows representative voltage maps demonstrating high and low proportions of low voltage, prior to removal of these structures, in patients mapped in AF and SR.

Prior to each blood sample aspiration, the contents of the femoral or intra-cardiac sheath were aspirated and discarded to ensure no contaminants were present in the sample. The sheath was flushed with heparinised saline after aspiration. Aspirated blood was transferred to serum separator tubes and allowed to clot for a minimum of 30 minutes. Once dense clot had formed, tubes were centrifuged for 15 minutes at 1600g. Aliquots of the separated serum were transferred to sterile, non-pyrogenic Eppendorf tubes and stored at -70°C until analysis. Samples were thawed prior to analysis, so underwent only one freeze-thaw cycle. Biomarkers of fibrosis were analysed using commercially available enzyme-linked immunosorbent assay (ELISA) kits. Pro-collagen type III N-terminal peptide (PIIINP) and galectin-3 (Gal-3) were analysed using kits produced by Elabscience (Beijing, China). Type I collagen C-terminal telopeptide (ICTP) was analysed using kits produced by Cusabio Life Science (Wuhan, China). Kits were processed according to the manufacturer’s instructions, and serial dilutions of serum were used to determine the appropriate dilution factor for each biomarker. Standards of known concentration and serum samples were tested in duplicate. Serum concentrations were extrapolated from optical density readings using a 4-parameter logistic curve derived from the standards, with background correction using blank wells. Inter- and intra-assay coefficients of variation were <15%. %. Lower limits of detection were: ICTP = 25ng/ml, gal-3 = 0.156ng/ml, FGF-23 = 15.625pg/ml, PIIINP = 23.438 pg/ml.

Planned follow-up duration was 365 days. Arrhythmia recurrence was defined as symptomatic or asymptomatic atrial fibrillation, or atrial tachycardia, with a duration of longer than 30 seconds, diagnosed on 12 –lead ECG or Holter monitor. In patients who experienced no recurrence according this criterion at follow-up, 24 hour Holter monitoring was performed to screen for asymptomatic arrhythmia. Continuous monitoring (e.g. with implantable loop recorder) was not employed, as the study was designed to represent clinically relevant endpoints. Such monitoring is not recommended in routine clinical practice as, in most cases, only symptomatic recurrence drives further intervention.[7]

The primary endpoint was arrhythmia recurrence after a 60-day blanking period. However, to adjust for recurrences due to pulmonary vein re-connection (as opposed to atrial fibrosis or other cause) a secondary endpoint was defined. This endpoint takes into account the effect of multiple procedures, and was defined as *either* a recurrence of arrhythmia after one procedure with no further intervention planned, *or* a recurrence of arrhythmia after a repeat procedure. Therefore, patients with recurrence after a single procedure, but no recurrence after a repeat ablation were not included in the secondary endpoint.

Continuous data were examined for normality using the Shapiro-Wilks test. Normally distributed data is expressed as ‘mean ± standard deviation’. Non-parametric data is expressed as ‘median *(interquartile range)’*. Categorical data is expressed as ‘frequency (percentage%)’. Where possible, non-parametric data was transformed to normally distributed data using logarithmic or square-root transformation, or categorised if appropriate. Comparison between two groups was performed using Student’s T-test for normally distributed data, Mann-Whitney U test for non-parametric data, or chi-squared test for categorical data. To examine time-to-outcome data, univariate Cox regression analysis was performed, followed by multivariate analysis using selected variables of interest (see Results). In order to assess possible predictive value of the biomarkers and voltage data, ROC curves were generated. In order to achieve this the variables were converted into binary discriminators using various possible cutoffs (e.g. levels above the median, levels in the highest quartile etc.). The quoted area-under-the-curve figures for each variable represent the most predictive cutoff determined. Analysis was carried out using SPSS version 22.

To achieve 80% power to detect the difference in AF rhythm outcome based on biomarker levels, 20 AF recurrences would be required. Based on documented departmental recurrence rates, and allowing for participant dropout, the target for recruitment was therefore 90 patients.

## Results

**Fig 2:**
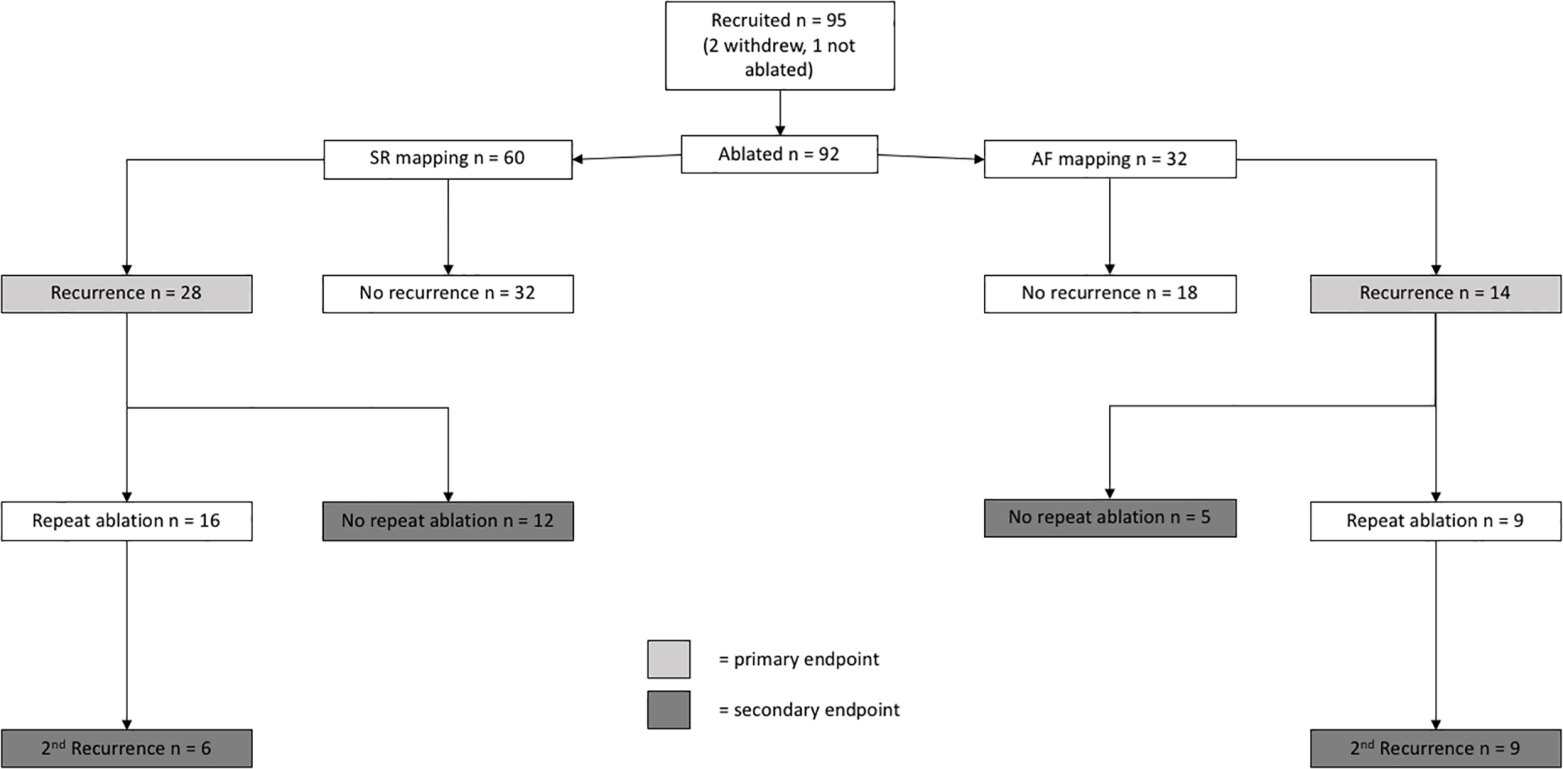
Study outline.

Fig 2 shows the patient recruitment and flow through the study. 98 ablation patients were screened. 3 patients failed screening due to meeting exclusion criteria. No patients refused to consent. 95 patients were therefore recruited into the ablation cohort. Two of these patients subsequently elected not to undergo ablation and were excluded. One procedure was abandoned due to pericardial bleeding after EP mapping and blood sampling, but before ablation. No further ablation was attempted, so this patient could not be included in outcome analysis. 60 patients were in sinus rhythm during atrial mapping, 32 were in AF. All patients had drug-refractory AF, defined by ongoing symptoms after therapy with at least 2 consecutive or concurrent agents, or unacceptable side-effects thereof.

Full presentation and analysis of baseline characteristics, including comparison with a non-AF control group, has been previously published.[9] Tables 1 and 2 show the baseline characteristics of the cohort, separated by the endpoints. Mean LA voltage was higher in those mapped in SR than in AF (1.3 V ± 0.6 V vs 0.8 V ± 0.4 V, p = 0.037). The findings of cross sectional analysis of the biomarkers levels within the cohort, at different sampling sites, and comparison with controls, has been published previously. In summary, after multivariate regression analysis, higher BMI was related to higher gal-3 (P<0.001), as was female sex (p = <0.001). Reduced LV ejection fraction (LVEF) was related to higher ICTP (p = 0.005). PIIINP levels were related to longer time since AF diagnosis (p = 0.003) and the presence of a history of cerebrovascular disease (p = <0.001). There was no significant association between any of the biomarkers and proportion of low voltage in the LA. There was no significant difference between levels of FGF-23 or PIIINP across sampling sites, so levels are quoted as a mean value of all four sampling sites. ICTP at the CS and gal-3 at the LA were shown to be significantly different from peripheral levels, so levels at these sites are shown in addition to the mean.

**Table 1:** Baseline characteristics according to primary endpoint (first procedure recurrence).

Characteristic	AF recurrence n = 42	No AF recurrence n = 50	P
**Age years**	56.0 *(22*.*9)*	60.1 *(17*.*48)*	0.956
**BMI kg/m**^**2**^	25.3 *(5*.*0)*	28.9 *(6*.*89)*	0.327
**Female sex**	11 (26.2%)	17 (34.0%)	0.417
**Hypertension**	13 (31.0%)	18 (36.0%)	0.610
**Diabetes Mellitus**	6 (14.3%)	3 (6.0%)	0.183
**IHD**	2 (4.8%)	3 (6.0%)	0.794
**Non-PAF**	15 (35.7%)	15 (30.0%)	0.560
**Time since 1**^**st**^ **AF diagnosis months**	34.3 *(33*.*4)*	24.3 *(53*.*85)*	0.420
**CHA**_**2**_**DS**_**2**_**VASc > = 2**	15 (35.7%)	20 (40.0%)	0.673
**Mean LA Pressure >11mmHg**	24 (57.1%)	32 (64.0%)	0.811
**Mean RA Pressure >6mmHg**	23 (54.8%)	20 (40.0%)	0.068
**LA volume / BSA >28 ml**	24 (57.1%)	27 (54.0%)	0.886
**LA diameter / BSA >23 mm**	5 (11.9%)	4 (8.0%)	0.538
**LV EDV / BSA >75 ml**	0 (0.0%)	2 (4.0%)	0.204
**LV EF <55%**	5 (11.9%)	3 (6.0%)	0.285
**Mean PIIINP pg/ml (n = 69)**	51.6 *(91*.*1)*	44.9 *(115*.*1)*	0.156
**Mean ICTP ng/ml (n = 79)**	329.4 *(190*.*1)*	300.0 *(373*.*0)*	0.121
**CS ICTP ng/ml (n = 76)**	297.5 *(209*.*2)*	331.7 *(253*.*6)*	0.314
**Mean gal-3 ng/ml (n = 81)**	30.8 *(75*.*4)*	24.5 *(40*.*0)*	0.510
**LA gal-3 ng/ml (n = 81)**	18.17 *(37*.*1)*	24.8 *(31*.*6)*	0.709
**Mean FGF-23 pg/ml (n = 33)**	32.6 *(45*.*5)*	50.5 *(62*.*8)*	0.313
**LA low voltage >30.0%, all participants (n = 92)**	15 (35.7%)	8 (16.0%)	**0.030**

**Table 2:** Baseline characteristics according to secondary endpoint.

Characteristic	Met secondary endpoint n = 32	Did not meet secondary endpoint n = 60	P
**Age years**	58.6 *(20*.*1)*	57.8 *(16*.*9)*	0.296
**BMI kg/m**^**2**^	26.9 *(9*.*6)*	28.9 *(7*.*4)*	0.861
**Female sex**	9 (28.1%)	19 (31.7%)	0.725
**Hypertension**	9 (28.1%)	22 (36.7)	0.409
**Diabetes Mellitus**	5 (15.6%)	4 (6.7%)	0.168
**IHD**	2 (6.3%)	3 (5.0%)	0.801
**Non-PAF**	12 (37.5%)	18 (30.0%)	0.465
**Time since 1**^**st**^ **AF diagnosis months**	34.4 *(29*.*6)*	32.7 *(34*.*8)*	0.641
**CHA**_**2**_**DS**_**2**_**VASc > = 2**	11 (34.4%)	24 (40.0%)	0.597
**Mean LA Pressure >11mmHg**	17 (53.1%)	39 (65.0%)	0.384
**Mean RA Pressure >6mmHg**	18 (56.3%)	25 (41.7%)	0.152
**LA volume / BSA >28ml**	20 (62.5%)	31 (51.7%)	0.261
**LA diameter / BSA >23 mm**	5 (15.6)	4 (6.6%)	0.151
**LV EDV / BSA >75 ml**	0 (0.0%)	2 (3.3%)	0.325
**LV EF <55%**	4 (12.5%)	4 (6.7%)	0.276
**Mean PIIINP pg/ml (n = 69)**	51.9 *(100*.*2)*	39.6 *(78*.*74)*	0.492
**Mean ICTP ng/ml (n = 79)**	290.8 *(267*.*2)*	333.7 *(344*.*9)*	0.114
**CS ICTP ng/ml (n = 76)**	305.7 *(163*.*2)*	328.4 *(275*.*8)*	0.829
**Mean gal-3 ng/ml (n = 81)**	36.1 *(75*.*4)*	24.1 *(35*.*3)*	0.938
**LA gal-3 ng/ml (n = 81)**	17.7 *(47*.*5)*	26.1 *(31*.*6)*	0.888
**Mean FGF-23 pg/ml (n = 33)**	34.5 *(29*.*6)*	46.8 *(60*.*7)*	0.808
**LA low voltage >30.0%, all participants (n = 92)**	11 (34.4%)	12 (20.0%)	0.129

**Fig 3:**
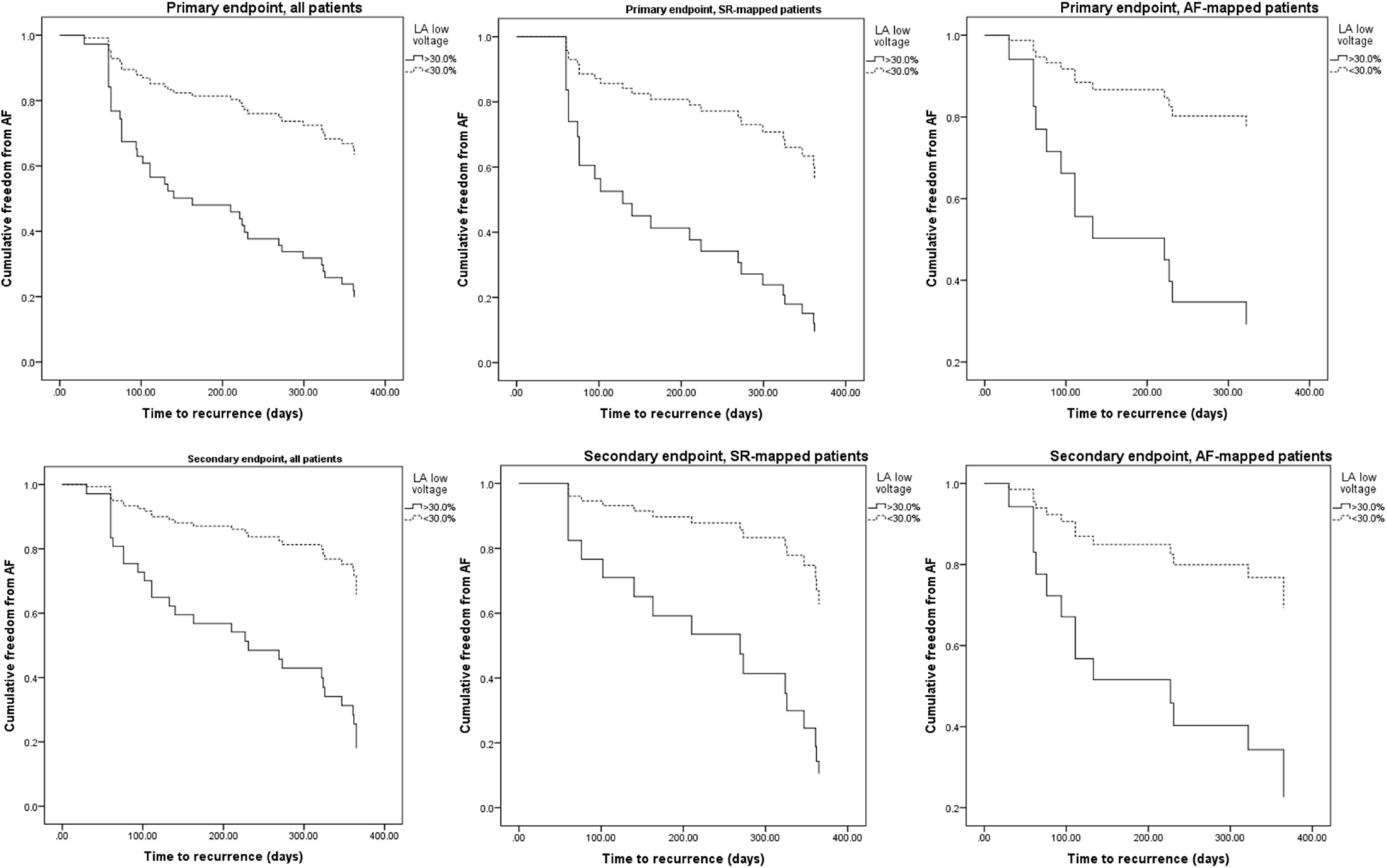
Freedom from AF assessed by each endpoint, separated by LA low voltage proportion >30.0%, mapped in SR and AF.

During the 365-day follow-up, 42 patients met the primary endpoint. 28 of these patients had been mapped in SR, 14 in AF. Comparison between the characteristics of those who met the primary endpoint and those who did not is shown in Table 1. For variables with clearly defined normal ranges (e.g. echocardiographic measurements), continuous data was categorized accordingly. For the biomarker levels, receiver-operator curves (ROC) were generated which revealed no predictive value (best area under the curve (AUC) for mean ICTP = 0.375, ICTP CS = 0.410; mean gal-3 = 0.478, gal-3 LA = 0.483; mean PIIINP 0.586; mean FGF-23 = 0.481). Further outcome analysis was therefore not carried out on the biomarkers. ROC for LA voltage (AUC = 0.653) suggested that values in the 4^th^ quartile (>30.0% low voltage) were stronger predictors of the endpoint, so data was categorised according to this. LA low voltage was analysed for those mapped in SR, AF and for the whole cohort. Those patients who met the primary endpoint had significantly higher proportion of LA low voltage tissue in the whole cohort (p = 0.030), and in those mapped in SR (p = 0.042), but not those mapped in AF (p = 0.178). This variable was therefore entered into a multivariate analysis with variables of interest, including predictors of AF recurrence published in other studies. These variables were age, body mass index (BMI), sex, LA volume, AF classification, AF duration (Table 3). Proportion of LA low voltage remained the only significant predictor variable in this analysis whether the patient was mapped in AF (hazard ratio 5.195, 95% confidence interval 1.032–26.141, p = 0.046) or SR (HR 4.323, 95%CI 1.337–13.982, p = 0.014). Fig 3, first row, shows cumulative freedom from AF, according to the primary endpoint, in the overall cohort, AF and SR-mapped patients. Curves are separated by 4^th^ versus combined other quartiles of LA low voltage proportion.

**Table 3:** Cox regression, LA low voltage proportion, primary endpoint.

	Hazard ratio (95% CI)	P
**LA low voltage >30.0%, all participants**	3.448 (1.626–7.313) *3*.*494 (1*.*600–7*.*633)*	0.001 ***0*.*002***
**LA low voltage >30.0%, SR during mapping**	4.471 (1.384–14.441) *4*.*323 (1*.*337–13*.*982)*	0.012 ***0*.*014***
**LA low voltage >30.0%, AF during mapping**	4.477 (1.167–17.170) *5*.*195 (1*.*032–26*.*141)*	0.029 ***0*.*046***

Italics = multivariate analysis. Variables entered into multivariate regression analysis: Age, body mass index, sex, LA volume / BSA, AF classification, AF duration—all were non-significant

As seen in Fig 2, there were 18 and 14 secondary endpoints in the SR and AF groups, respectively. Table 2 shows comparison of baseline characteristics. Again, none of the biomarker levels or clinical characteristics (including LA low voltage) were significantly different. LA low-voltage was again subjected to Cox regression analysis, the results of which are seen in Table 4. The same variables were used for multivariate regression. LA low voltage remained a significant predictor whether the LA was mapped in SR (HR 10.375, 95%CI 2.049–52.538, p = 0.005) or AF (HR 6.200, 95%CI 1.194–32.194, p = 0.030). Due to the reduction in number of endpoints, confidence intervals were wider for this endpoint definition.

**Table 4:** Cox regression, LA low voltage proportion, secondary endpoint.

	Hazard ratio (95% CI)	P
**LA low voltage >30.0%, all participants**	4.084 (1.944–8.580) *5*.*000 (2*.*042–12*.*244)*	<0.001 ***<0*.*001***
**LA low voltage >30.0%, SR during mapping**	4.832 (1.503–15.532) *10*.*375 (2*.*049–52*.*538)*	**0.008** ***0*.*005***
**LA low voltage >30.0% AF during mapping**	3.565 (1.050–12.106) *6*.*200 (1*.*194–32*.*194)*	0.042 ***0*.*030***

Italics = multivariate analysis. Variables entered into multivariate regression analysis: Age, body mass index, sex, LA volume / BSA, AF classification, AF duration—all were non—significant

Comparison of low voltage LA proportion in those patients with arrhythmia recurrence but no repeat procedure, and those who had a repeat procedure, revealed no difference (27.8% ± 12.9% vs. 27.6% ± 9.2%, p = 0.967). This comparison was carried out to look for evidence of selection bias when planning repeat procedures.

## Discussion

None of the biomarkers assessed in this study were predictive of arrhythmia recurrence after AF ablation, as assessed by either of the endpoints. Proportion of low voltage in the LA was predictive of arrhythmia recurrence after a single procedure, whether the LA was mapped in SR or AF. This effect remained when repeat procedures were considered.

The background of these biomarkers, and the reasons for choosing to study them, have been previously published.[9]

This study found that none of the biomarkers selected was predictive of AF recurrence. This therefore adds to the conflicting evidence regarding PIIINP, ICTP and Gal-3.[10–13] FGF-23 has not been studied in this context before. The challenge when using biomarkers to assess a pathology is specificity. In this instance, the goal is to assess *cardiac* fibrosis, specifically, LA fibrosis. As discussed, while these biomarkers have been shown to be involved in cardiac pathology both *in vitro* and *in vivo*, they are far from exclusively so. Therefore, the blood levels are liable to be affected by fibrosis elsewhere in the body. For example, we have previously shown that ICTP and gal-3 have lower intra-cardiac than peripheral levels, with no significant difference in the cases of FGF-23 and PIIINP.[9] Therefore, it appears that if any AF-related cardiac processes are indeed causing release of these biomarkers into the bloodstream, systemic fibrosis masks this in peripheral blood.

A further challenge is the large degree of scatter exhibited in the levels of the biomarkers. This may be in part due to limitations of the ELISA technique, but may also again reflect the diversity of processes in which these biomarkers are involved. Such degrees of variation make prediction on an individual patient basis challenging.

The findings of this study refute our hypothesis and suggest that, for these biomarkers at least, there is no clinical utility in the prediction of arrhythmia recurrence, and therefore they are no aid to patient selection.

The findings of this study agree with the larger 2005 study by Verma et al. which found that low voltage areas in the LA were the only predictor of AF recurrence after multivariate analysis.[5] The method of assessing the presence of low voltage differs between the two studies. Rather than defining discrete areas of scarring within the atrium and treating this as a binary variable, this study shows that the approach of quantifying low voltage values as a proportion of the LA endocardium is sufficient to have a predictive effect for rhythm outcome. The study has also shown that this effect is present using a high-density mapping catheter without the need for intra-cardiac ultrasound. The use of a contact force-sensing ablation catheter during the ablation was sufficient to validate the shell. The upper voltage threshold of 0.5mV has been suggested by several studies as a cutoff between normal and abnormal tissue.[5, 14] It should be noted that these studies mapped patients in sinus rhythm. The lack of voltage reference criteria in AF-mapped patients could be considered a limitation of this study. However, despite clear differences in overall LA voltage values in AF-mapped patients compared to SR-mapped patients, the same threshold of 0.5mV showed utility in predicting AF recurrence in both groups. While EP mapping is not a useful tool in the selection of patients for first-time ablation procedures, such voltage information may be useful to the operator when considering repeat procedures. Therefore, as approximately one third of patients who undergo ablation are in AF at the start of the case, it is useful to know that voltage analysis remains relevant; if such a patient were to experience recurrence, the previous voltage mapping data could be one of a number of factors influencing the decision to return for further ablation. These findings support our hypothesis, although it should be noted that due to the small numbers of AF-mapped patients, the confidence interval for this predictive effect is wide. A larger study would be useful to confirm this effect.

Most studies have used recurrence after first-time ablation as an endpoint. However, this does not allow for the effect of PV re-connection due to tissue healing, or resolution of transient causes of electrical block such as edema. Recurrences due to these phenomena are not a result of fibrosis. Therefore, we also used a secondary endpoint that took multiple procedures into account. The significant predictive effect of LA voltage was present for both endpoints, although confidence intervals for the hazard ratio were wide due to the lower number of patients meeting the secondary endpoint. Although the analysis of voltage was carried out after the ablation procedure, operators could not be blinded to the standard CARTO or Velocity visual voltage map during cases, so to assess whether later selection for repeat ablation had been influenced by voltage maps from the index procedure, we compared voltage values of recurrent AF patients who underwent redo procedures with those who did not, and found no difference. This suggests that selection bias did not play a significant role.

The study population represents a ‘real-world’ AF ablation population with minimal exclusion criteria, and the findings should therefore be generalizable. The recurrence rate is broadly consistent with other studies examining outcomes after AF ablation in populations including persistent AF patients.[15, 16] Caution should be employed when interpreting the findings involving FGF-23 as results were not available for a large proportion of the patients, however we have included this in the results as this biomarker has not been studied in this context previously. To confirm the predictive value of voltage mapping in AF using the secondary endpoint, a larger trial is required. Finally, although Holter monitoring was used to detect asymptomatic recurrences, undetected asymptomatic recurrences cannot be completely ruled out. The lack of relationship between voltage and any baseline characteristics may be related to the smaller numbers in this study compared to others.

## Conclusions

ICTP, PIIINP, Gal-3 and FGF-23 are not predictive of AF recurrence after RF ablation.

The presence of low voltage tissue within the atrium, assessed using a semi-automated technique, is predictive of AF recurrence when the atrium is mapped in SR or AF. Further development of this may allow operators to improve assessment of the likelihood of AF recurrence in their patients.

## Supporting information

S1 supporting information Anonymised raw study data. (XLSX)
